# PROTOCOL: Policies and interventions to remove gender‐related barriers to girls’ school participation and learning in low‐ and middle‐income countries: A systematic review of the evidence

**DOI:** 10.1002/cl2.1047

**Published:** 2019-09-05

**Authors:** Erica K. Chuang, Barbara S. Mensch, Stephanie R. Psaki, Nicole A. Haberland, Meredith L. Kozak

**Affiliations:** ^1^ Population Council New York New York

## 1 BACKGROUND

### 1.1 The problem: gender equality in schooling and the gendered school environment

Successful policies and programs are directly tied to the quality of the evidence informing them, but too often scarce resources are invested in education interventions that are not evidence‐based. Low‐ and middle‐income countries have made enormous progress in expanding primary school enrollment since the 1990s. Yet questions remain about the causes and consequences of continued gender gaps in school enrollment, progression, skill acquisition and school quality, as well as the best approaches to promoting broader outcomes like ambition, agency, cognitive ability and critical thinking skills.

Despite UNESCO's declaration that gender parity had been achieved globally in primary education by 2014 (UNESCO, 2016), a recent analysis of trends over 10 years in 43 countries between 1997 and 2007 (Time 1) and 2008 and 2014 (Time 2; Psaki, McCarthy, & Mensch, 2018) revealed that, in many low‐ and middle‐income countries:

● Progress in girls’ educational attainment has stagnated; of the 43 countries, only three made substantial progress in both female attainment and gender parity.
● Female disadvantages persist in access to school, especially in countries with low enrollment; at Time 2 fewer girls than boys were enrolled in primary school in 12 countries.elf‐awareness, self‐management, social awareness, relationship skills, responsible
● Once enrolled, girls tend to complete as many grades as boys; in the 12 countries in which fewer girls than boys completed primary school, the gap in attainment was completely due to gaps in school enrollment.
● Gender parity in attainment may mask gender‐specific barriers to retention such as unplanned pregnancy for girls and work pressures for boys.
● Gender parity in attainment does not necessarily translate into gender parity in learning; in nine of 23 countries with data on literacy, boys were more likely than girls to have acquired basic literacy skills after having completed primary school.

Much has been written about the importance of education for girls, particularly the strong association between educational attainment and reproductive, maternal and child health in developing countries (Lutz & Kebede, 2018; Mensch, Chuang, Melnikas, & Psaki, 2019; Psaki, Chuang, Wilson, Melnikas, & Mensch, 2019). Researchers have argued that, in addition to developing academic skills and competencies that facilitate interaction with health bureaucracies, education is a socializing institution that potentially gives rise to more equitable gender attitudes and greater autonomy, which are likely gained through school‐based experiences and shifting expectations about the future (Jejeebhoy, 1995; Lloyd and Mensch, 1999). Better‐educated women are said to be more able to act on health knowledge, and navigate health institutions, thereby improving child health (Joshi, 1994; LeVine, LeVine, Schnell‐Anzola, Rowe, & Dexter, 2011; Smith‐Greenaway, 2013). Education is thought to change reproductive preferences by increasing the opportunity cost of childbearing for women who, by virtue of skills gained, have expanded opportunities to engage in the labour market (Jejeebhoy, 1995). Education is also believed to alter norms about family size and the role of children such that the cost of children is higher among the better educated who are more likely to invest in quality rather than quantity of offspring (Becker, 1981).

In addition to the health benefits, considerable evidence exists about the economic returns to education for women. Indeed, analyses have indicated that overall, rates of return to female education in terms of lifetime earnings net of current costs are higher than those for male education. Moreover, the advantage for females has increased over time (Psacharopoulos and Patrinos, 2018).

Despite widespread agreement on the importance of education for girls, key gender‐related barriers remain to ensuring that girls enter school on time, complete at least a secondary education, and gain basic literacy and numeracy skills. Although some barriers to schooling, such as cost, may be fairly consistent across settings, gender‐related barriers to schooling are also likely to reflect local gender norms, as well as other structural and policy‐related factors. For example, in some settings, the school environment may be detrimental with teachers reported to have negative attitudes towards girls and low expectations of their academic ability, reflecting broader gender norms, and potentially undermining girls’ achievement (Lloyd & Mensch, 1999). Evidence has also emerged of sexual, physical and psychological abuse of girls at school (DevTech, 2004; EFA Global Monitoring Report, UNESCO, & UNGEI, 2015; Leach, Dunne, & Salvi, 2014; Leach, Fiscian, Kadzamira, Lemani, & Machakanja, 2003), which may reflect, in part, local attitudes and practices related to the acceptability of corporal punishment and other forms of violence (Barasa, Wamue‐Ngare, & Wanjama, 2013; Crooks, Scott, Wolfe, Chiodo, & Killip, 2007; Leach & Humphreys, 2007). In addition, compared to their male peers, in some contexts, girls are considerably more likely to experience early marriage and childbearing, with consequences for school continuation (Psaki, 2016). Beyond the social effects of early marriage and childbearing, policies barring adolescent mothers and pregnant girls from school are also likely to contribute to gender differences in educational attainment (UNESCO, 2018). Finally, when financial resources are limited, parents with traditional gender role attitudes may prefer to keep sons in school rather than daughters (Lloyd & Young, 2009).

Despite a considerable literature documenting gender‐related barriers, gaps in knowledge exist regarding the degree to which interventions to reduce gender‐related barriers to schooling in low‐ and middle‐income countries are effective in improving education outcomes for girls. Further, while not within the scope of this study, even in settings where gender gaps in school enrollment and completion have narrowed, questions remain as to whether those improvements will translate into longer‐term benefits, including labour market participation (World Bank, 2008) and improved health outcomes (Mensch et al., 2019; Psaki, et al., 2019).

### 1.2 The interventions

**Figure 1:**
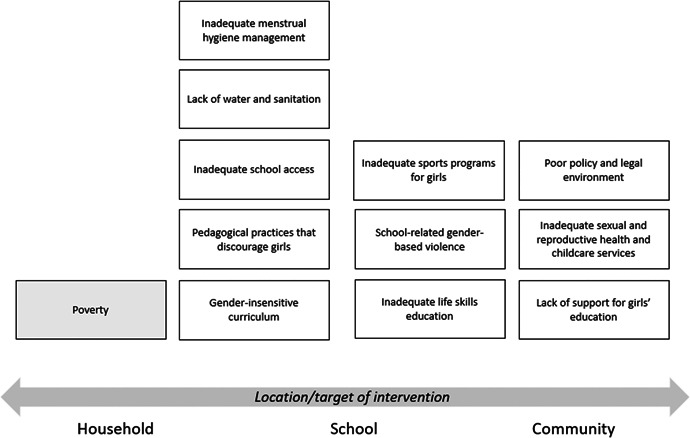
Location of interventions to reduce gender‐related barriers

One of the goals of this review is to identify the variety of interventions targeting gender‐related barriers to girls’ schooling that have been evaluated. We list below commonly perceived barriers and describe ways those barriers may potentially affect girls’ schooling. The extent to which these barriers exist likely varies between settings, and to our knowledge has not been studied comprehensively. While it would be premature to describe all such interventions and components in advance of the literature search, we provide examples of interventions that potentially address each gender‐related school barrier listed (see Figure 1).^1^ Next to each gender‐related barrier we indicate whether the interventions listed address a deficit of physical, social or human capital. Borrowing from the economics literature, we categorize interventions by whether the primary instruments or tools of an intervention aim to directly improve the physical, social, or human capital of girls. Capital is generally defined as “durable produced items that are in turn used as productive inputs for further production” (Samuelson & Nordhaus, 2011). For the purposes of this study, we designate the production output as gains in education. Physical capital, as the name suggests, consist of tangible inputs to production and can include “structures (and) equipment” such as schools, roads and automobiles (Samuelson & Nordhaus, 2011). Social capital is defined as a girl's family, friends and associates, and the direct interaction between the girl and these actors (Woolcock, 2001). Interventions that fall under this category would include teacher training of gender‐responsive pedagogy, community‐wide information interventions to promote girls’ schooling, and interventions to encourage interactions between girls, such as sports programs. Human capital is defined as “the skills (a) labor force possesses”. Interventions that affect human capital are “investments in people” and can include inputs such as implementation of new curricula, hiring more teachers in all‐girl schools, and sexual and reproductive health services provision (Goldin, 2014). We understand and fully expect there to be interventions that span more than one of these categories, for example, hiring female teachers, which would increase a girl's interaction with potential female role models (social) as well as boost access to schooling (human).

1. Inadequate school access (physical)—Access to school is one of the most basic barriers to education. At the primary level, students must sometimes travel long distances to and from school each day. At the secondary level, many rural communities lack secondary schools, so students have to find boarding opportunities or live with friends or relatives. In both cases, these barriers may be particularly challenging for girls due to concerns about safety and the appropriateness of adolescent girls living outside their parents’ homes. Interventions that address school access will do one (or more) of the following:
  a. Increase the number of schools available to girls, for example, through building of community schools.
  b. Increase the availability of school transport for girls, for example, through provision of bicycles or school buses or “walking bus” programs (where school children are chaperoned by parents and/or community volunteers with the adults acting as a “driver” and “conductor” along a set route).
  c. Increase boarding opportunities for girls at school.
  d. Provide flexible school schedules.
2. Inadequate sexual and reproductive health and childcare services (human)—The availability and accessibility of quality reproductive health services, such as those providing HIV testing, treatment and care, may increase access to and performance in school for adolescent girls by reducing unwanted pregnancies and sexually transmitted infections. In addition, childcare services for adolescent girls who have given birth may enable the young mother to return to school. Interventions that address inadequate sexual and reproductive health and childcare services for girls will do one (or more) of the following:
  a. Improve the quality of existing sexual and reproductive health services for adolescents, for example by training providers on how to provide family planning counselling services to this population.
  b. Introduce, or extend access to, sexual and reproductive health services, either in school or in another setting.
  c. Introduce, or extend access to, childcare services for young mothers, either at school or in another setting in the community.
3. Inadequate life skills (human)—Provision of life skills education, such as gaining knowledge about reproductive health, or building core social and emotional skills, may equip young people, including girls, with the knowledge and skills to remain in school and improve school performance. For example, strong self‐management and decision‐making skills may enable students to spend time studying outside of school, and critical consciousness and agency may enable girls in particular to advocate for their right to stay in school in settings where child marriage is common. Interventions that address inadequate life skills will do one (or more) of the following:
  a. Improve girls’ sexual and reproductive health knowledge, including knowledge about prevention and treatment of HIV/AIDS.
  b. Build empowerment and psycho‐social skills (resilience/social and emotional skills).
4. Gender insensitive curriculum (human)—Textbooks and educational materials that portray women in traditional gender roles may undermine girls’ learning, academic performance and retention in school. In addition, there is some evidence that girls’ schools have inferior science equipment compared to boys’ schools, which may contribute to girls’ poorer performance in STEM subjects. Interventions that address a gender insensitive curriculum will do one (or more) of the following:
  a. Provide gender‐sensitive teaching materials.
  b. Modify textbooks to ensure that gender stereotyping is eliminated.
  c. Ensure that girls have equal access to learning materials.
5. Pedagogical practices that discourage girls (human/physical)—Calling on boys more than girls in class and systematic differences between boys and girls regarding time devoted to classroom chores may affect girls’ interest in school and their academic performance. Gender training of teachers to encourage positive attitudes towards the intellectual capacity of girls, supportive teaching practices that engage girls, and the hiring of female teachers may all enhance girls’ school experiences. Interventions that address pedagogical practices that discourage girls will do one (or more) of the following:
  a. Train teachers in gender‐responsive pedagogy.
  b. Recruit, train and retain female teachers.
  c. Create book, math and science clubs for girls.
  d. Establish clinics or tutoring sessions for girls, for example in STEM subjects.
6. Lack of support for girls’ education (social)—Sensitization of students, parents, teachers and wider community regarding girls’ schooling and equitable gender norms may improve girls’ attitudes towards, and performance in, school. Teacher, parent and community attitudes about innate abilities of and appropriate roles for girls may discourage girls from learning and lead to premature school dropout. Normative beliefs, even if not based on evidence, likely affect girls’ academic expectations, school performance and behaviours. Interventions that improve support for girls’ education will do one (or more) of the following:
  a. Change community knowledge and attitudes about the value of girls’ education, for example through community‐wide information campaigns on the benefits of girls’ schooling.
  b. Change community attitudes about domestic responsibilities for girls that affect school participation.
  c. Change community, parental and girls’ norms and behaviours in order to reduce child marriage.
  d. Change teachers’ and school administrators’ attitudes related to girls’ education, for example through information and training programs.
7. Inadequate menstrual hygiene management (physical/human)—The inability to manage menstruation whether due to insufficient menstrual materials, fear of leaking, concern about odour and stained clothing, lack of water, soap and private toilet facilities to change, wash and dry protection materials, as well as limited access to pain relief medication, may affect school attendance and enrollment for pubescent girls. Interventions that improve the ability of girls to manage menstruation will do one (or more) of the following:
  a. Provide free or subsidized sanitary products (sanitary pads and/or underwear).
  b. Provide free or subsidized analgesics for physical discomforts (cramps and headaches).
  c. Educate girls and others about menstruation and menstrual hygiene management.
8. Lack of water and sanitation (physical)—Inadequate water, sanitation and hygiene (WASH) in schools is said to result in adverse health and cognitive outcomes. Specifically, limited access to water and dirty and inadequate toilet facilities may increase absenteeism and school dropout due to diarrhoeal and gastrointestinal diseases. The lack of safe, private and single sex toilets may be a source of embarrassment in addition to effecting educational and health outcomes. Interventions that improve access to clean water and sanitation facilities will do one (or more) of the following:
  a. Provide new sources of water at schools by, for example, drilling more boreholes.
  b. Construct hand‐washing stations at schools.
  c. Construct/improve school toilets.
  d. Provide single sex toilets.
9. School‐related gender‐based violence (social)—SRGBV is defined by UNESCO and UNGEI as: “Acts or threats of sexual, physical or psychological violence occurring in and around schools, perpetrated as a result of gender norms and stereotypes, and enforced by unequal power dynamics” (UN Women, 2016; UNGEI, 2015). In addition to its effects on physical and mental health, SRGBV is said to undermine learning and increase absenteeism. Interventions that address SRGBV will do one (or more) of the following:
  a. Modify school policies and practices to create a safer environment, for example through the development and implementation of codes of conduct and safety policies.
  b. Change students’ knowledge and attitudes about violence, for example by developing and implementing anti‐violence curricula/activities for students.
  c. Change teacher and school administrator behaviour, for example by training school personnel on prevention and reporting of violence.
10. Poor policy/legal environment (social)—The policy and legal environment, including awareness of existing policies, may affect girls’ access to and performance in school in numerous direct and indirect ways, including those that affect all students (e.g., elimination of school fees), and those that affect only girls (return to school policies following childbirth). Interventions that address the policy and legal environment will do one (or more) of the following:
  a. Raise awareness about existing laws/policies among students, teachers and parents, for example, those allowing pregnant girls to remain in school and return to school after childbirth.
  b. Develop or promote new laws/policies, such as increasing the number of compulsory years of schooling.
11. Inadequate sports programs for girls (physical/social)—If sports facilities or equipment at school are absent for girls by comparison to boys, opportunities to develop physical skills, practice leadership, and engage in teamwork will be limited, which may affect girls’ views about appropriate gender roles and even performance in school. Interventions that improve girls’ ability to play sports will do one (or more) of the following:
  a. Institute school policies to ensure that girls get equal access to sports’ facilities.
  b. Provide sports equipment for girls at school.
  c. Offer new sports programs for girls, or extend existing programs to include girls, at school.
12. Other—We provide an “other” category in order to include papers analysing interventions that address a gender‐related barrier not listed above. Such interventions would address situations in which the authors assert that there are assets, activities or facilities for which a gender difference in access exists. For example, in resource‐constrained settings girls may be less likely to get access to food, or iron supplementation or preventive health care or bed nets and lack of or limited access may negatively affect education outcomes for girls. For a paper to be included in this category, the authors would have to state explicitly that a gender differential in access exists, to girls’ detriment. Because it may not be entirely clear whether a paper addresses a gender‐related barrier that is not listed above—for example, in situations where the authors do not state a priori that the intervention addresses such a barrier but then conclude, based on their findings, that a gender‐related barrier exists—procedures for contacting the authors, and resolving ambiguities, are included in Section 3.

Note that by including papers in this “other” category we may overstate the positive effect of the interventions examined because we will not be investigating papers that assess the effectiveness of the same interventions in settings where the authors do not discuss a gender difference or barrier. Nonetheless, we think it is worth including such papers in order to have as exhaustive a set of interventions that address gender‐related barriers in education. In addition, it should be noted that while interventions aimed at poverty reduction are common and address an important barrier to girls’ schooling, they are outside the scope of this review.

Three interventions provide a better understanding of the types of programs likely to be assessed in this review. Based on the above typology we have noted the components in each.

1. A randomized controlled trial (RCT) among 8th grade school girls in Lusaka, Zambia evaluating a program that provided training in negotiation skills (on such topics as rebuffing unwanted sexual advances from older men and asking parents to pay school fees) for girls via six, 2‐hr sessions. To investigate the mechanisms underlying the treatment effects, two alternative randomized interventions were also conducted, a safe space program that provided girls with a female mentor and all‐female space but did not teach the negotiation curriculum, and a short intervention that provided girls with information about the returns to education (in addition to information on HIV prevention). Girls were randomized into three groups: (a) control, (b) safe space and (c) negotiation group. The negotiation group was further cross‐randomized with information treatment. A “horse race” was run between the negotiation treatment and the safe space treatment to determine whether the negotiation curriculum had effects beyond any empowerment or aspirations. The outcome measures were paid school fees, took junior secondary exam, performance on national exam in math and English, school attendance, school dropout and pregnancy. Cox regression models were estimated for school dropout and conventional regression models were estimated for other outcomes (Ashraf, Bau, Low, & McGinn, 2018).
2. An intervention in Burkina Faso, known as the BRIGHT program, that provided an integrated package of “girl‐friendly” education interventions in primary schools including separate latrines for boys and girls, a borehole for clean water, take home rations for girls conditional on 90% attendance, an information campaign for parents on the potential benefits of girls’ education, placement of female teachers in program schools and gender sensitivity training for teachers and ministry officials. Other infrastructure elements were also included. The program was evaluated using a regression discontinuity design. Girls’ enrollment and test scores in math and French were assessed as were gender differences in these outcomes (Kazianga, Levy, Linden, & Sloan, 2012, 2013).
3. An RCT in rural northwest Afghanistan to provide village‐based schools. The goal of the intervention was to encourage parents to send their children to school, particularly girls who are often not permitted by parents to enroll if they must travel far to attend. While the schools had basic supplies, new facilities were not constructed; the schools were located in spaces provided by the villages. The outcome measures were enrollment status and score on a math and language test with questions taken from government textbooks. Effects were estimated for boys and girls. Regression models were estimated; two‐stage least squares models were estimated to assess test scores with enrollment instrumented using treatment assignment (Burde & Linden, 2013).

These examples illustrate that many interventions to address gender‐related barriers to girls’ schooling consist of multiple components. In the absence of factorial evaluation designs, it will not be possible to entirely determine whether a particular component is efficacious or whether it is the package of components working, perhaps, synergistically that produce an effect. However, it is informative to include multicomponent interventions within our review in order to illustrate the potential need for studies that employ factorial designs in cases where there is no current evidence that delineates effects based on the separate components of an intervention.

### 1.3 How the interventions might work

**Figure 2:**
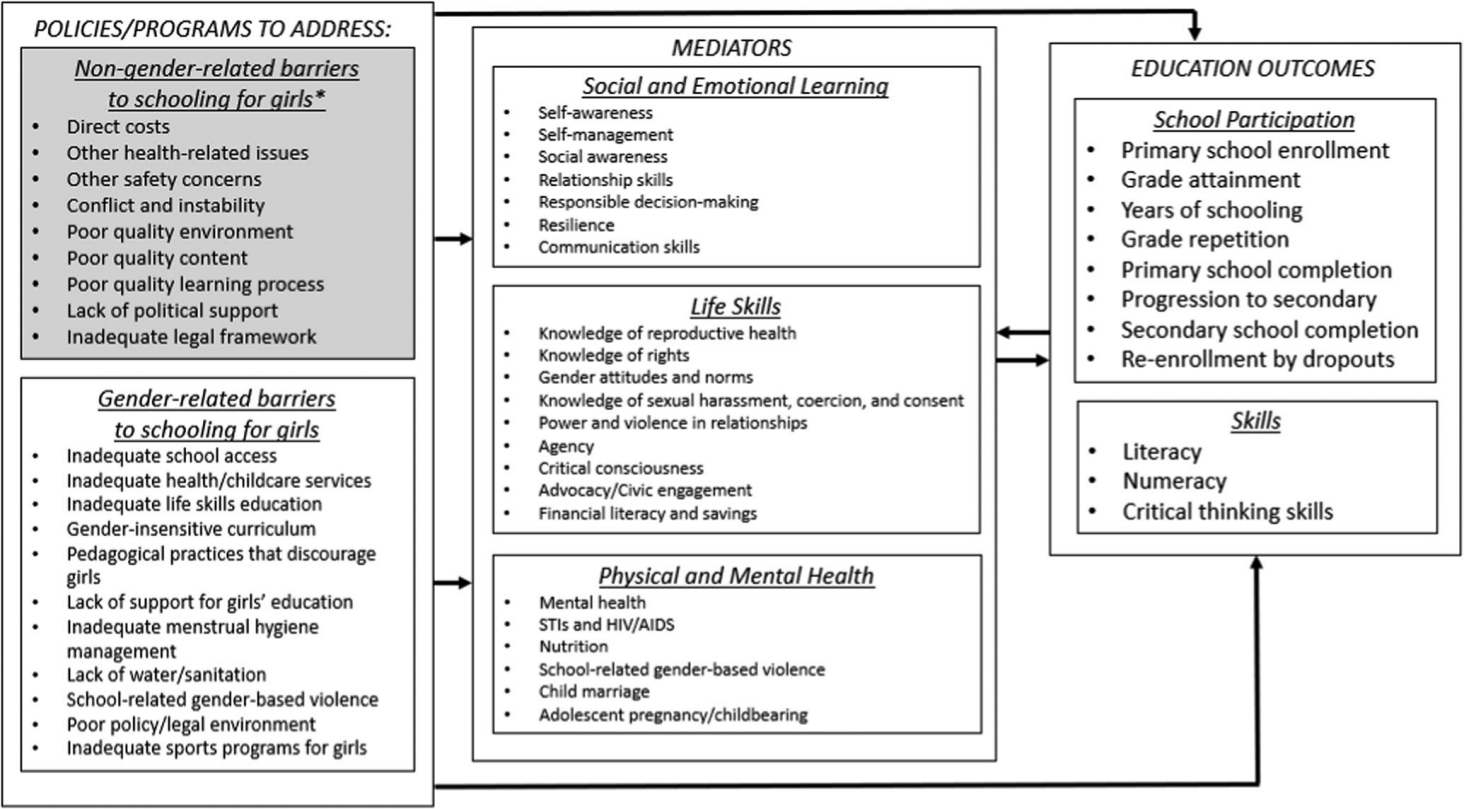
Conceptual framework linking barriers to school for girls to outcomes

**Figure 3:**
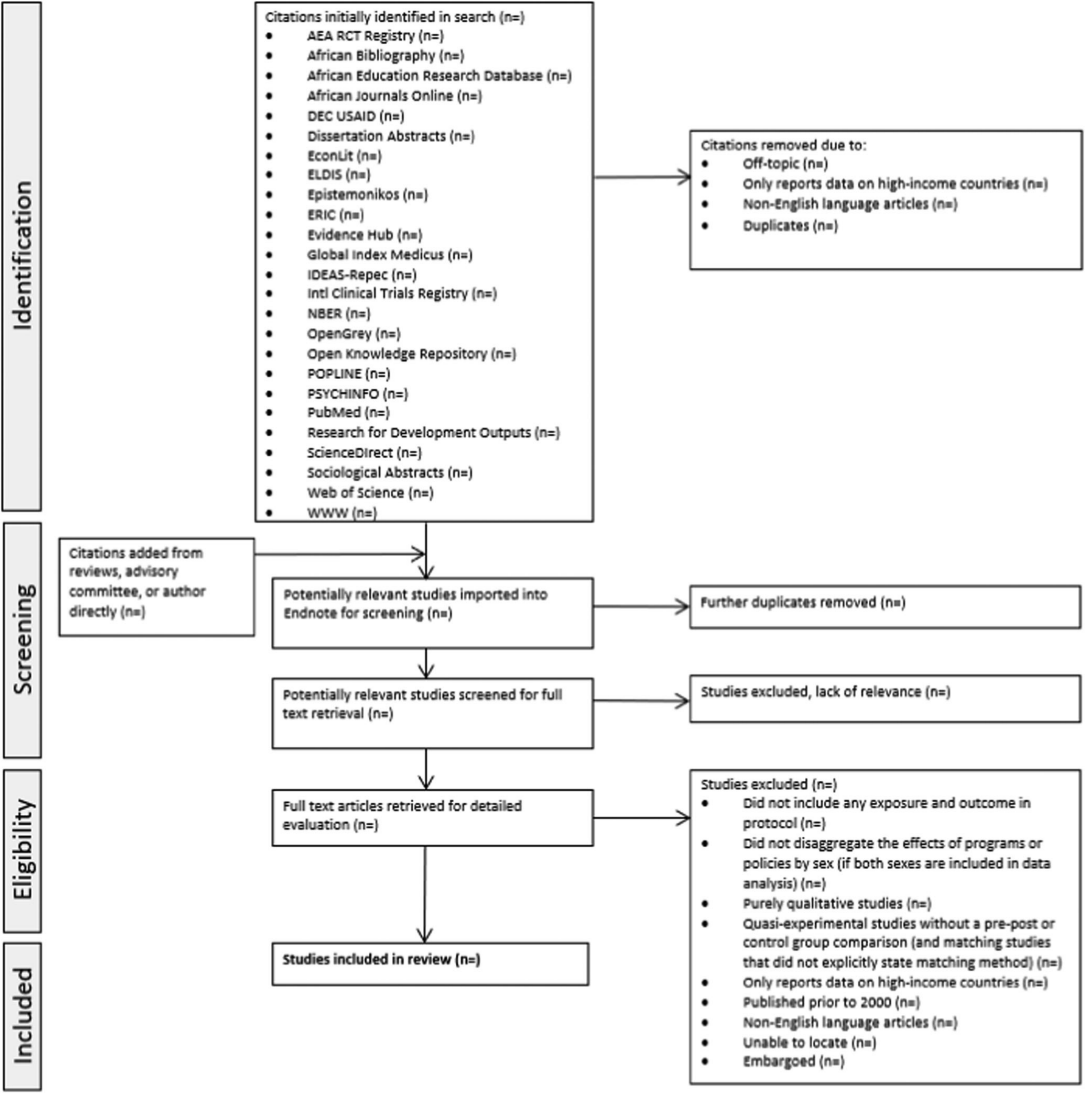
PRISMA flow diagram

The conceptual model above (Figures 2 and 3) lists types of gender and non‐gender‐related barriers to schooling for girls that included interventions might address, as well as the mediators and education outcomes that are potentially affected by those barriers.

As discussed, the gender‐related barriers to schooling vary between settings, as do the appropriate interventions to address those barriers. However, many of those interventions share similar underlying logic. For example, interventions may target parents’ attitudes about the value of girls’ education using different approaches, including sharing information about employment opportunities for women or making a rights‐based argument through community meetings. Given parents’ roles in deciding whether their children will attend school, and providing financial resources to support schooling, such interventions might have a direct effect on school enrollment and retention. More indirectly, teacher training programs that equip teachers with the skills to understand and address the learning needs of both girls and boys may lead to more active participation of girls in the classroom, leading to stronger communication skills and transformed gender attitudes for girls, and subsequently leading to improved grade attainment and literacy for girls.

The primary outcomes of interest in this review are focused on education and skills. However, the relationship between mediators in our conceptual framework and education outcomes is likely bidirectional, and we may identify studies that shed light on that relationship in both directions. Although listed as mediators, understanding the effects of policies and interventions on social and emotional learning, life skills and physical and mental health are important questions in their own right. However, to maintain a reasonable scope for this review, our inclusion criteria are focused primarily on studies measuring education and skills outcomes. Therefore, our results will provide only a partial picture of the evidence on the effects of policies and interventions on social and emotional learning, life skills and physical and mental health.

### 1.4 Justification for review

#### 1.4.1 Informing practice and policy

Sustainable Development Goal (SDG) 4, adopted as part of a broader global development agenda in 2015, focuses on expanding access to quality education and lifelong learning, including by eliminating gender disparities in schooling (target 4) by 2030. This renewed agenda builds on previous global commitments, such as the Millennium Development Goals, which pledged to achieve education for all children by 2015. However, while the vast majority (96%) of the world's countries are signatories to the 1979 Convention on the Elimination of All Forms of Discrimination against Women (CEDAW), 27 have entered reservations pertaining to gender equality in education (UNESCO, 2018). Further, despite numerous global policy commitments, there exist countries with policies preventing girls from completing their schooling. For example, as of June 2018, Tanzania, Sierra Leone, and Equatorial Guinea have banned girls who are pregnant or have already given birth from attending government‐run schools (Human Rights Watch, 2018).

Though gender inequalities in education in many LMICs persist, examples exist of policies aimed directly at addressing gender‐related barriers to education in these settings. For example, Uganda passed legislation in the last decade updating curricula in early childhood and primary schools to be more gender‐sensitive (Nabbuye, 2018). The Philippines implemented the Anti‐Bullying Act in 2013 to curb school‐related violence, specifically referencing and condemning bullying based on gender (UNESCO, 2015). In addition, the Female Secondary School Stipend Programme in Bangladesh and the Bourses Maman programme in Mali are both nationally implemented conditional cash transfer programs that address financial obstacles that inhibit household investment in girls’ schooling (UNESCO, 2018).

The need for better‐informed policies and programs is evident. This systematic review will investigate whether interventions to eliminate gender‐related barriers improve education and other outcomes for girls. The goal is to identify which programs are most effective at addressing these barriers.

#### 1.4.2 Prior education reviews in low‐ and middle‐income countries

A considerable number of reviews, many systematic, have been conducted that assess the evidence regarding the effectiveness of interventions to improve education outcomes in developing countries. To date, the most comprehensive review has been conducted by the International Initiative for Impact Evaluation (3ie), which published a systematic review and meta‐analysis entitled, “Interventions for improving learning outcomes and access to education in low‐ and middle‐income countries” in 2015 (Snilstveit et al., 2015), along with an evidence gap map highlighting 21 previous or ongoing systematic reviews in education. In summarizing the findings, the authors noted that programs either improve school participation or learning but rarely impact both. “Strong and consistent evidence” was found for cash transfer programs in affecting participation and “structured pedagogy programs”, defined as “evidence‐based curricula and instructional approaches” for affecting learning.

In contrast to the proposed review, the 3ie review did not explicitly focus on gender‐related barriers. As such, for the most part, the interventions analysed did not specifically target girls and disaggregation by sex was either not a component of the reviews or, when included, was not a focus of the analysis. Only when assessing interventions specifically targeting girls, for example, conditional cash transfers and sex segregated toilets (see, e.g., Ganimian & Murnane 2016), were analyses focused on girls. This reflects the fact that most education evaluations do not examine heterogeneity in effects by sex (two noteworthy exceptions are Duflo, Dupas, & Kremer, 2011; Lucas, McEwan, Ngware, & Oketch, 2014).

This review is investigating several of the same or similar intervention categories as the Snilstveit et al. (2015) 3ie review. However, this review has an overt gender focus, aiming to investigate interventions that explicitly target girls, for example, hiring of female teachers or interventions that the conceptual framework suggests will have a disproportionate effect on girls, for example, improving transportation to school. To clarify the distinction between this review and the 3ie review, below we list intervention categories in the 3ie review and the comparable categories with a gender focus in our review:

- School‐based health (3ie) versus “Sexual and reproductive health and childcare services.
- Teacher hiring (3ie) versus “Recruit, train and retain female teachers”.
- Providing materials (3ie) versus “Provide gender‐sensitive teaching materials, and Modify textbooks to ensure that gender stereotyping is eliminated.”
- Teacher training (3ie) versus “Train teachers in gender‐responsive pedagogy”.

Several reviews focusing explicitly on girls’ education have also been conducted recently, most notably those by Sperling and Winthrop (2015) and Unterhalter (2014). While valuable contributions to the body of evidence on what works in girls’ education, compared to the general education reviews (e.g., by 3ie), reviews on girls’ education have tended to be less systematic in their search strategy, used less rigorous criteria for ratings of study quality or inclusion, and have not provided quantitative syntheses of effects of different approaches. Although these reviews have considered how programs may affect girls and boys differently—an analysis that has been largely lacking in the more general education reviews—they have also tended to focus on addressing school‐level barriers, overlooking girls who have never attended school or dropped out prematurely.

In addition, several systematic reviews have been conducted looking at the effects of water and sanitation facilities and interventions to improve menstrual hygiene management on educational and psycho‐social outcomes for girls. Lack of clean water, lack of private and safe toilets, and inadequate and unhygienic menstrual supplies are said to be a source of diarrhoeal and gastrointestinal diseases, reproductive tract infections (bacterial vaginosis and candidiasis), embarrassment and stress. However, despite assertions that inadequate sanitation facilities and inability to manage menstruation are a major cause of girls’ absenteeism, dropout and poor learning outcomes, very few empirical studies support these claims (Birdthistle, Dickson, Freeman, & Javidi, 2011; Hennegan & Montgomery, 2016; Jasper, Le, & Bartram, 2012). Papers from these prior systematic reviews that meet our inclusion criteria will be included in our review, as will papers published since these reviews were conducted (Birdthistle et al., 2011; Hennegan & Montgomery, 2016; Jasper et al., 2012).

In order to identify programs most effective for girls’ access and learning, the World Bank has conducted a secondary analysis of a 3ie database containing existing systematic review data in addition to other studies in their own database, with the goal of comparing interventions aimed at girls with general interventions; sex‐disaggregated data have been requested from authors in order to complete the analyses (Evans & Yuan, 2018). The analysis focuses on two outcomes for girls, access and learning, and attempts to answer the question of which approach—girl‐targeted or those including both girls and boys—is more effective. The Evans and Yuan review does not include outcomes such as life skills and other social and emotional learning (SEL), norm‐changing outcomes and secondary education interventions.

#### 1.4.3 Contribution of this review

This review departs from those that already exist in that we aim to identify if, and to what extent, policies and interventions designed to address gender‐related barriers to girls’ school participation and learning are effective in enrolling girls in schools, retaining them as students, ensuring quality learning and helping prepare them for the transition to work and healthy adulthoods. Studies that analyse these relationships may take the form of RCTs or quasi‐experimental analyses of programs and policies, as detailed below.

As the education community pursues expanded global goals on educational attainment, lifelong learning and gender equality, the need to align high‐quality evidence with policies and programs is more pressing than ever. This review is designed to fill a gap in existing evidence on what works to address gender‐related barriers to schooling, which may exist at the individual, household, community, school or policy levels. Specifically, we will explore whether interventions and policies designed to remove gender‐related barriers to girls’ school participation and learning are effective when it comes to enrolling girls in schools, retaining them as students, ensuring quality learning and helping prepare them for the transition to work and healthy adulthoods. Through the proposed systematic review, we will identify interventions and policies to remove gender‐related barriers to girls’ school attendance and learning for which:

- the evidence is strong (either in support or against);
- the evidence is weak or non‐existent; and
- gaps in evidence exist but the approaches are promising such that further investment in program evaluation is desirable.

The contribution of this systematic review is to bridge the gap between the most rigorous reviews, and those that use a gender‐grounded conceptual framework, to produce clear guidance on the state of evidence of what works in girls’ education.

## 2 OBJECTIVES

The objective of this systematic review is to summarize and assess evaluations of policies and interventions to address gender‐related barriers that are believed to undermine girls’ school participation and learning. We define gender‐related barriers as factors that prevent girls from enrolling, attending, fully participating and/or learning in school. Barriers may exist at the individual, household, community, school or policy levels and we draw on the work of UNICEF, UNESCO, the Global Partnership for Education and the United Nations Girls’ Education Initiative on barriers to girls’ education to collate what barriers may exist (Albright, 2016; Antoninis et al., 2018; Secretariat, 2016; UNICEF, 2002).

The primary research question we will address is: What is the effect of interventions to eliminate gender‐related barriers to girls’ education on girls’ primary and secondary school participation and learning outcomes in low‐ and middle‐income countries? Specifically, what is the effect of interventions to address gender‐related barriers on:

- Enrollment and grade attainment for girls? (Grade attainment includes years of schooling, highest grade completed, completion of primary school, transitioning from primary to secondary school, completion of secondary school and re‐entry for girls who have dropped out.)
- School attendance for girls?
- Grade repetition for girls?
- Learning for girls? (This includes academic skills, particularly literacy and numeracy, as well as “skills and mindsets” such as agency and critical thinking.)

Secondary questions we will address are:

1. Which pathways operate in effective interventions to eliminate, or mitigate the effects of, gender‐related barriers to schooling?
2. Given the expectation that there will be considerable heterogeneity in effect sizes, what study characteristics account for the variation? That is, what are the potential moderators at the aggregate and study level that explain the variability in the relationship between the interventions to reduce gender‐related barriers and education outcomes for girls?

## 3 METHODOLOGY

The following section outlines our methodological approach to the review. These methods are largely based on the Cochrane Handbook, *Practical Meta‐Analysis* by Mark Lipsey and David B. Wilson, and *Research Synthesis and Meta‐Analysis: A Step‐by‐Step Approach* by Harris Cooper (Cooper, 2015; Higgins & Green, 2011; Lipsey & Wilson, 2001).

### 3.1 Criteria for including and excluding studies

In order to assess the current evidence on interventions to address gender‐related barriers to schooling for girls, studies published prior to 2000 will be excluded. We will not exclude studies based on language.

#### 3.1.1 Types of study designs

Studies that attempt to control for endogeneity^2^ through one or more of the following methods will be eligible for inclusion^3^:

- RCTs (longitudinal data, or postintervention data for studies with large sample sizes^4^).
- Regression discontinuity (longitudinal or cross‐sectional data).
- Instrumental variables analysis (longitudinal or cross‐sectional data).
- Other quasi‐experimental studies with either:
  - a matched comparison group where matching procedure described (e.g., nearest‐neighbour matching, propensity score matching), or
  - a comparison group where quasi‐treatment and quasi‐control groups are either stratified or tested for balance, both based on more than one sociodemographic characteristic that are justified by background literature.
- Interrupted time series (longitudinal data).

While RCTs are largely considered the gold standard when evaluating the effect(s) of a given intervention, exogenous changes in policies and exposures that act as quasi‐experiments can also provide valuable insight when evaluating said effect(s), particularly when paired with quantitative methods that attempt to control for endogeneity. Thus, studies reporting on both RCTs and quasi‐experiments are eligible for inclusion. Articles that utilize study designs outside of those listed above will be excluded, notably:

- RCTs that only report postintervention data from a small‐scale intervention.
- Quasi‐experimental studies without a pre‐post or quasi‐control group comparison.
- For studies that employ matching, no formal matching method was stated.

#### 3.1.2 Types of participants

While the focus of the review is on girls living in low‐ and middle‐income countries as defined by the World Bank (2019), we do not explicitly exclude studies that include boys in the study population as long as the results for boys and girls are disaggregated. Studies that do not disaggregate results by sex will be excluded.

#### 3.1.3 Types of interventions/exposures

Below is the list of example interventions and the gender‐related barriers they are associated with, taken from Section 1. Studies identified in the search must analyse the results of interventions/exposures that target a gender‐related barrier to education, otherwise they will be excluded^5^:

1. Inadequate school access (physical)—Access to school is one of the most basic barriers to education. At the primary level, students must sometimes travel long distances to and from school each day. At the secondary level, many rural communities lack secondary schools, so students have to find boarding opportunities or live with friends or relatives. In both cases, these barriers may be particularly challenging for girls due to concerns about safety and the appropriateness of adolescent girls living outside their parents’ homes. Interventions that address school access will do one (or more) of the following:
  a. Increase the number of schools available to girls, for example, through building of community schools.
  b. Increase the availability of school transport for girls, for example, through provision of bicycles or school buses or “walking bus” programs (where school children are chaperoned by parents and/or community volunteers with the adults acting as a “driver” and “conductor” along a set route).
  c. Increase boarding opportunities for girls at school.
  d. Provide flexible school schedules.
2. Inadequate sexual and reproductive health and childcare services (human)—The availability and accessibility of quality reproductive health services, such as those providing HIV testing, treatment and care, may increase access to and performance in school for adolescent girls by reducing unwanted pregnancies and sexually transmitted infections. In addition, childcare services for adolescent girls who have given birth may enable the young mother to return to school. Interventions that address inadequate sexual and reproductive health and childcare services for girls will do one (or more) of the following:
  a. Improve the quality of existing sexual and reproductive health services for adolescents, for example by training providers on how to provide family planning counselling services to this population.
  b. Introduce, or extend access to, sexual and reproductive health services, either in school or in another setting.
  c. Introduce, or extend access to, childcare services for young mothers, either at school or in another setting in the community.
3. Inadequate life skills (human)—Provision of life skills education, such as gaining knowledge about reproductive health, or building core social and emotional skills, may equip young people, including girls, with the knowledge and skills to remain in school and improve school performance. For example, strong self‐management and decision‐making skills may enable students to spend time studying outside of school, and critical consciousness and agency may enable girls in particular to advocate for their right to stay in school in settings where child marriage is common. Interventions that address inadequate life skills will do one (or more) of the following:
  a. Improve girls’ sexual and reproductive health knowledge, including knowledge about prevention and treatment of HIV/AIDS.
  b. Build empowerment and psycho‐social skills (resilience/social and emotional skills).
4. Gender insensitive curriculum (human)—Textbooks and educational materials that portray women in traditional gender roles may undermine girls’ learning, academic performance and retention in school. In addition, there is some evidence that girls’ schools have inferior science equipment compared to boys’ schools, which may contribute to girls’ poorer performance in STEM subjects. Interventions that address a gender insensitive curriculum will do one (or more) of the following:
  a. Provide gender‐sensitive teaching materials.
  b. Modify textbooks to ensure that gender stereotyping is eliminated.
  c. Ensure that girls have equal access to learning materials.
5. Pedagogical practices that discourage girls (human/physical)—Calling on boys more than girls in class and systematic differences between boys and girls regarding time devoted to classroom chores may affect girls’ interest in school and their academic performance. Gender training of teachers to encourage positive attitudes towards the intellectual capacity of girls, supportive teaching practices that engage girls, and the hiring of female teachers may all enhance girls’ school experiences. Interventions that address pedagogical practices that discourage girls will do one (or more) of the following:
  a. Train teachers in gender‐responsive pedagogy.
  b. Recruit, train and retain female teachers.
  c. Create book, math and science clubs for girls.
  d. Establish clinics or tutoring sessions for girls, for example in STEM subjects.
6. Lack of support for girls’ education (social)—Sensitization of students, parents, teachers and wider community regarding girls’ schooling and equitable gender norms may improve girls’ attitudes towards, and performance in, school. Teacher, parent and community attitudes about innate abilities of and appropriate roles for girls may discourage girls from learning and lead to premature school dropout. Normative beliefs, even if not based on evidence, likely affect girls’ academic expectations, school performance and behaviours. Interventions that improve support for girls’ education will do one (or more) of the following:
  a. Change community knowledge and attitudes about the value of girls’ education, for example through community‐wide information campaigns on the benefits of girls’ schooling.
  b. Change community attitudes about domestic responsibilities for girls that affect school participation.
  c. Change community, parental and girls’ norms and behaviours in order to reduce child marriage.
  d. Change teachers’ and school administrators’ attitudes related to girls’ education, for example through information and training programs.
7. Inadequate menstrual hygiene management (physical/human)—The inability to manage menstruation whether due to insufficient menstrual materials, fear of leaking, concern about odour and stained clothing, lack of water, soap and private toilet facilities to change, wash, and dry protection materials, as well as limited access to pain relief medication, may affect school attendance and enrollment for pubescent girls. Interventions that improve the ability of girls to manage menstruation will do one (or more) of the following:
  a. Provide free or subsidized sanitary products (sanitary pads and/or underwear).
  b. Provide free or subsidized analgesics for physical discomforts (cramps and headaches).
  c. Educate girls and others about menstruation and menstrual hygiene management.
8. Lack of water and sanitation (physical)—Inadequate water, sanitation and hygiene (WASH) in schools is said to result in adverse health and cognitive outcomes. Specifically, limited access to water and dirty and inadequate toilet facilities may increase absenteeism and school dropout due to diarrhoeal and gastrointestinal diseases. The lack of safe, private and single sex toilets may be a source of embarrassment in addition to effecting educational and health outcomes. Interventions that improve access to clean water and sanitation facilities will do one (or more) of the following:
  a. Provide new sources of water at schools by, for example, drilling more boreholes.
  b. Construct hand‐washing stations at schools.
  c. Construct/improve school toilets.
  d. Provide single sex toilets.
9. School‐related gender‐based violence (social)—SRGBV is defined by UNESCO and UNGEI as: “Acts or threats of sexual, physical or psychological violence occurring in and around schools, perpetrated as a result of gender norms and stereotypes, and enforced by unequal power dynamics” (UN Women, 2016; UNGEI, 2015). In addition to its effects on physical and mental health, SRGBV is said to undermine learning and increase absenteeism. Interventions that address SRGBV will do one (or more) of the following:
  a. Modify school policies and practices to create a safer environment, for example through the development and implementation of codes of conduct and safety policies.
  b. Change students’ knowledge and attitudes about violence, for example by developing and implementing anti‐violence curricula/activities for students.
  c. Change teacher and school administrator behaviour, for example by training school personnel on prevention and reporting of violence.
10. Poor policy/legal environment (social)—The policy and legal environment, including awareness of existing policies, may affect girls’ access to and performance in school in numerous direct and indirect ways, including those that affect all students (e.g., elimination of school fees), and those that affect only girls (return to school policies following childbirth). Interventions that address the policy and legal environment will do one (or more) of the following:
  a. Raise awareness about existing laws/policies among students, teachers and parents, for example those allowing pregnant girls to remain in school and return to school after childbirth.
  b. Develop or promote new laws/policies, such as increasing the number of compulsory years of schooling.
11. Inadequate sports programs for girls (physical/social)—If sports facilities or equipment at school are absent for girls by comparison to boys, opportunities to develop physical skills, practice leadership and engage in teamwork will be limited, which may affect girls’ views about appropriate gender roles and even performance in school. Interventions that improve girls’ ability to play sports will do one (or more) of the following:
  a. Institute school policies to ensure that girls get equal access to sports’ facilities.
  b. Provide sports equipment for girls at school.
  c. Offer new sports programs for girls, or extend existing programs to include girls, at school.
12. Other—We provide an “other” category in order to include papers analysing interventions that address a gender‐related barrier not listed above. Such interventions would address situations in which the authors assert that there are assets, activities or facilities for which a gender difference in access exists. For example, in resource‐constrained settings girls may be less likely to get access to food, or iron supplementation or preventive health care or bed nets and lack of or limited access may negatively affect education outcomes for girls. For a paper to be included in this category, the authors would have to state explicitly that a gender differential in access exists, to girls’ detriment. Because it may not be entirely clear whether a paper addresses a gender‐related barrier that is not listed above—for example, in situations where the authors do not state a priori that the intervention addresses such a barrier but then conclude, based on their findings, that a gender‐related barrier exists—procedures for contacting the authors, and resolving ambiguities, are included in Section 3.

Multicomponent interventions where one of the components is addressing gender‐related barriers will be included. Interventions and policies under investigation may take place at the primary or secondary levels or may be non‐formal for school‐aged young people.

For quantitative analyses, interventions/exposures must adhere to the following criteria in order to be grouped together:

- The intervention targets at least one common barrier to girls’ schooling.
- The intervention targets at least one common type of capital input, that is, physical, social or human capital.
- The level of implementation of the intervention is the same (individual, household, school, hospital/clinic, other community level and other).

More information about quantitative analyses are presented under Section 3.4.1, and will apply to both single and multicomponent interventions and exposures. Extraction of information for each of the above points will take place during the data extraction phase, described further in Section 3.3.2.

#### 3.1.4 Types of outcome measures

Studies that report results for the primary outcomes listed below will be included. Studies that do not include any of the primary outcomes below in their analyses will be excluded. Outcomes of interest are to be measured in all comparison groups whether pre/post or intervention/control. Note that the measures listed for each outcome below are how they are commonly assessed. Studies will not be excluded if they do not measure the outcomes in the way we outline below.

#### 3.1.5 Primary outcomes

The outcomes outlined below are general examples that fall under each construct and are relevant to studies that analyse individual‐level data; as noted, there may be other ways to measure the same construct that have not been accounted for and will be included. Some measures (e.g., grade attainment and years of schooling) are similar enough to combine in the same meta‐analysis. However, other outcomes (e.g., grade repetition and absenteeism) measure inherently different phenomena even though they both fall under the same umbrella outcome. In the latter case, we will not combine these in the same meta‐analysis. For more information on the quantitative synthesis, see Section 3.4.1.

1) Enrollment (limited to primary and secondary school)
  1. Grade attainment (e.g., continuous, where every unit increase is equal to an additional grade the individual progressed to).
  2. Years of schooling (e.g., continuous, where every unit increase is equal to an additional year of schooling).
  3. Enrollment in primary school (e.g., dichotomous, =1 if enrolled, =0 if otherwise).
  4. Enrollment in secondary school (e.g., dichotomous, =1 if enrolled, =0 if otherwise).
2) Retention
  1. Grade repetition (e.g., dichotomous, =1 if repeated a grade, =0 if otherwise).
  2. Primary school completion (e.g., dichotomous, =1 if completed primary school, =0 if otherwise).
  3. Progression to secondary school (e.g., dichotomous, =1 if attended secondary school =0 if otherwise).
  4. Secondary school completion (e.g., dichotomous, =1 if completed secondary school, =0 if otherwise).
  5. Re‐enrollment in school among dropouts (e.g., dichotomous, =1 if dropped out and re‐enrolled in school, =0 if otherwise).
  6. Absenteeism (e.g., continuous, number of school days the student did not attend in the last week).
3) Learning and transition to adulthood
  1. Academic skills (literacy and numeracy) during and after leaving school (e.g., dichotomous, =1 if able to read a paragraph prompted by the data collector, =0 if otherwise).
  2. Critical thinking skills (e.g., Test of Science Critical Thinking [TSCT]; Mapeala & Siew, 2015).

#### 3.1.6 Secondary outcomes

For the purposes of this study, we are interested in the secondary outcomes below as potential mediators through which interventions aimed at addressing gender‐related barriers may affect girls’ schooling. Lack of inclusion of any of the secondary outcomes below will not prevent studies from being included, but those that analyse the outcomes below without also analysing any of the primary outcomes listed above will be excluded.

##### 3.1.6.1 Attitudes/abilities

1) Social and Emotional Learning (CASEL core competencies: self‐awareness, self‐management, social awareness, relationship skills, responsible decision‐making (CASEL; https//casel.org)) as well as:
  - Resilience (e.g., Resilience Scale for Adolescents [READ]; von Soest, Mossige, Stefansen, & Hjemdal, 2010).
  - Communication skills (e.g., Communication Scale; Barkman & Machtmes, 2002).
2) Gender attitudes and norms (e.g., GEM Scale; Pulerwitz & Barker, 2008).
3) Power and violence in relationships (e.g., Sexual Relationship Power Scale; Pulerwitz, Gortmaker, & DeJong, 2000).
4) Agency (e.g., General Self Efficacy Scale; Schwarzer & Jerusalem, 1995).
5) Critical consciousness (e.g., Critical Consciousness Scale; Diemer, Rapa, Park, & Perry, 2017).
6) Advocacy/civic engagement (e.g., Sociopolitical Control Scale for Youth; Peterson, Peterson, Agre, Christens, & Morton, 2011).

##### 3.1.6.2 Knowledge

1) Knowledge of sexual and reproductive health (e.g., dichotomous, =1 if respondent has ever heard of a male condom, =0 if otherwise).
2) Knowledge of rights (e.g., dichotomous, =1 if respondent knows legal age of marriage, =0 if otherwise).
3) Understanding of sexual harassment, coercion and consent (e.g., dichotomous, =1 if respondent knows that a person consenting to a sexual experience can say no to the same experience in the future, =0 if otherwise).

##### 3.1.6.3 Physical and mental health

1) Mental health (e.g., PHQ‐9; Kroenke & Spitzer, 2002).
2) STIs and HIV/AIDS (e.g., dichotomous, =1 if HIV positive, =0 if otherwise).
3) School‐related gender‐based violence (e.g., dichotomous, =1 if experienced bullying from a peer in the playground this school year, =0 if otherwise).
4) Child marriage (below age 18; e.g., dichotomous, =1 if married below age 18, =0 if otherwise).
5) Adolescent pregnancy/childbearing (below age 18; e.g., dichotomous, =1 if given birth before age 18, =0 if otherwise).

##### 3.1.6.4 Resource access

1) Financial literacy and savings (e.g., dichotomous, =1 if respondent saves money in a bank account, =0 if otherwise).
2) Nutrition measures, such as incidence/prevalence of anaemia (e.g., dichotomous, =1 if anaemic, =0 if otherwise).

#### 3.1.7 Duration of follow‐up

The authors will not exclude studies based on duration of follow‐up but will note duration in the data extraction table.

#### 3.1.8 Types of settings

Studies that report on the primary outcomes listed above using data from low‐ and middle‐income countries at the time of the intervention/exposure, as defined by the World Bank, will be included. Studies that only report on outcomes from high‐income countries, as defined by the World Bank, will be excluded.

### 3.2 Search strategy

The following databases will be searched electronically:

**Table:** 

Database name	Platform	Web address
AEA RCT Registry	AEA	https://www.socialscienceregistry.org/trials/search?
Africa Bibliography	Cambridge Univ Press	https://africabibliography.cambridge.org/
African Education Research Database	REAL Centre, ESSA	https://essa‐africa.org/node/501?action=searchadvanced
African Journals Online	AJOL	https://www.ajol.info/index.php/index/search
DEC USAID	USAID	https://dec.usaid.gov/dec/content/AdvancedSearch.aspx?
Dissertation Abstracts	ProQuest	https://search.proquest.com/genre=dissertations+%26+theses&sid=ProQ:ProQuest+Dissertations+%26+Theses+Global
EconLit	ProQuest	https://search.proquest.com/genre=article&sid=ProQ:ProQ%3Aeconlit
ELDIS	IDS	https://www.eldis.org/search
Epistemonikos	Epistemonikos	https://www.epistemonikos.org/en/advanced_search
ERIC	EBSCOhost	https://search.ebscohost.com/login.aspx?direct=true&db=eric
Evidence Hub	3ie	http://www.3ieimpact.org/evidence‐hub
Global Index Medicus	WHO Global Health Library	http://www.globalhealthlibrary.net
IDEAS‐Repec	IDEAS‐Repec	https://ideas.repec.org/search.html
Intl Clinical Trials Registry	WHO ICTRP	http://apps.who.int/trialsearch/
NBER	NBER	https://www.nber.org/papers.html
OpenGrey	INIST‐CNRS	http://www.opengrey.eu/search/
Open Knowledge Repository	WB	https://openknowledge.worldbank.org/
POPLINE	JHUCCP	https://www.popline.org/advancedsearch
PsychINFO	EBSCOhost	https://search.ebscohost.com/login.aspx?direct=true&db=psyh
PubMed	NLM.NIH	https://www.ncbi.nlm.nih.gov/pubmed/advanced
Research for Development Outputs	DFID R4D	https://www.gov.uk/dfid‐research‐outputs
ScienceDirect	Elsevier	https://www.sciencedirect.com/search?
Sociological Abstracts	ProQuest	https://search.proquest.com/genre=article&sid=ProQ:ProQ%3Asocabs
Web of Science	EBSCO/Reuters	https://search.ebscohost.com/login.aspx?direct=true&db=web

Grey literature will be identified using the databases listed above from DEC, ELDIS, OpenGrey and R4D. Studies published in all languages will be included. Additional unpublished/ongoing studies will be identified through searches of websites of specific organizations identified in the search to be key resources. These organization websites will include: Center for Global Development, CARE, CEDPA, High‐Quality Technical Assistance for Results (HEART), International Center for Research on Women (ICRW), J‐PAL (Poverty Action Lab), Population Council, UNESCO, UNGEI and UNICEF. References from these websites will be reported in a general category (World Wide Web, i.e., WWW). Please note that additional organizations we identify through the search may be added to this list.

The search strategy documented in Appendix Section A will be used to conduct searches through ERIC and will be adapted to conform to the search functions of the other databases. Reference lists and bibliographies in relevant review articles and reports of systematic reviews found in the search will also be combed in order to identify additional articles eligible for inclusion. Reviewers will also contact relevant researchers and organizations identified through the search as key resources in the field to locate additional articles eligible for inclusion.

### 3.3 Details of study coding categories

#### 3.3.1 Article screening

Articles will be identified through searching the databases listed in the search strategy shown in Appendix Section A. Initial screening will remove duplicate entries. After duplicates are removed, each title and abstract will be screened by two reviewers based on the inclusion criteria documented above through Covidence. In the case of any disagreements, the two reviewers will meet to discuss the article. If they do not resolve their disagreement, a third team reviewer (E. K. C. or B. S. M.) will be used to settle disagreements regarding study inclusion. Prior to the full text review, a sample of ten randomly selected articles will be jointly reviewed by the project team and any disagreements will be discussed in order to establish that interpretation of the content of each article is similar for the two reviewers. Randomized selection of articles will be performed in Stata 15. Once the final set of abstracts is agreed upon, full text will be linked to each article. Full text will be reviewed by two reviewers, following the same screening and disagreements resolution procedures as the title and abstract review.

#### 3.3.2 Data extraction

Data extraction, along with intervention grouping, will be completed by two reviewers through an online tool (Google Forms); instructions for the form can be found in Appendix Sections D–F. The data extraction form has been designed in consultation with the Cochrane Handbook (Higgins & Green, 2011) and adapted from Psaki et al. and Mensch et al. (2019). Where data are missing that could determine the inclusion eligibility of a study, such as interventions that may fall in the “Other” category, or effect size conversion, reviewers will contact the study author(s) to request the relevant information; three attempts to contact the author(s) will be made within one month. If the author(s) do not respond or does not provide the relevant information within one month of the first date of contact, then the study will be excluded from quantitative synthesis but included in narrative synthesis. Tables 1 and 2 provide an example subset of the data extraction form meant to provide ease of readership and may change depending on journal requirements. The complete data extraction form will be included as a part of supplementary materials on Dataverse.

**Table 1:** Characteristics of included studies

Title	Authors	Type of publication	Countr(ies)	Dates of data collection	Study design	Analysis method(s)	Study population	Age range	Sample size	Male results included
										
										

**Table 2:** Results

Study	Country	Age range	Sample size	Attrition/response rate (if RCT)	Intervention/exposure	Intervention/exposure measure (Dichotomous vs. continuous)	Frequency and duration of exposure	Single versus multicomponent	Components included	Education outcome	Outcome measure (dichotomous vs. continuous)	Standardized effect size (95% CI)	Intervention cost
													

#### 3.3.3 Risk of bias assessment

We will perform an assessment of risk of bias in both experimental and quasi‐experimental studies adapted from RoB 2 (Higgins et al., 2016) for randomized studies and ROBINS‐I (Sterne et al., 2016) for nonrandomized studies. Risk of bias will be assessed by two reviewers. Any disagreements will be discussed and if the initial two reviewers cannot agree, a third reviewer will assist in resolving any disputes. As a part of the nonrandomized studies tool, we include methods‐specific criteria from Psaki et al. (2019), which were adapted from Baird, Ferreira, Özler, and Woolcock (2013). While RCTs are generally considered the gold standard for identifying causal impact, even they may be subject to threats to validity (e.g., differential loss to follow‐up and selective reporting of outcomes). Given the larger body of literature that utilizes quasi‐experimental methods, our risk of bias assessment tool will incorporate criteria for both experimental and quasi‐experimental studies. The full assessment of risk of bias tool is presented in Appendix Section B. The relevant section of the risk of bias assessment tool will be applied to each included study at the time of data extraction.

### 3.4 Quantitative analyses

We will first provide a narrative summary of characteristics of included studies, including country, type of publication, sample size, the type of analysis conducted, estimation procedures used, and our assessment of risk of bias. We will then provide quantitative summaries of the findings grouped by intervention/exposure and outcomes, converting each result into a partial correlation. Effect size conversion, method of analysis, assessment of publication and small study bias, as well as sensitivity analyses procedures are provided below in more detail.

#### 3.4.1 Statistical procedures and conventions

We expect that the vast majority of effect sizes presented in the included set of studies will be in the form of unstandardized regression coefficients. All effect sizes will be converted into partial correlations for the purposes of this quantitative analysis. If information cannot be obtained from either the article(s) or the authors of included studies to convert the results into partial correlations, these results will not be included in the quantitative analysis but will be included in the narrative summary. Due to the anticipated prevalence of covariates used in the models within our expected pool of included studies, we choose to convert to partial correlations as it represents the relationship between two variables, controlling for (i.e., “partialling out”) covariates, and range in value from −1 to 1 (Aloe & Thompson, 2013; Aloe, 2014). Corresponding 95% confidence interval (CI) will be calculated for all outcomes if not already provided, and estimates presented in either tabular form or a forest plot. We include equations to convert effect sizes, standard errors, and 95% CI in Appendix Section C, which are drawn from Aloe and Thompson (2013), The Campbell Collaboration (Polanin & Snilstveit, 2016), and formulas developed in consultation with David B. Wilson for the Psaki et al. and Mensch et al. (2019) reviews.^6^ Conversion equations will be chosen based on the requisite information to convert effect sizes available.^7^ We will use one conversion equation for all effect sizes grouped by the criteria below where possible and appropriate,^8^ defaulting to the equation where all studies within each group and model type provide the same information necessary for conversion.

Effect sizes of studies will be grouped and analysed based on the following study characteristics:

- Intervention/Exposure (grouping criteria taken from Section 3.1.3):
  - The intervention targets at least one common barrier to girls’ schooling.
  - The intervention targets at least one common type of capital input, that is, physical, social or human capital.
  - The level of implementation of the intervention is the same (individual, household, school, hospital/clinic, other community level and other).
- Randomized versus Quasi‐experimental study.
- Education outcome.
- Unit of analysis.

We will not quantitatively combine the results of randomized and quasi‐experimental studies into the same meta‐analyses, nor will we combine interventions that fall into different categories in Section 3.1.3 list. Results from multicomponent interventions and exposures where multiple barriers are targeted may be reported in more than one meta‐analysis, based on the barriers they share and common level of implementation.

In addition, some studies may be subject to unit of analysis error, where the unit of analysis does not match the unit of allocation (Higgins & Green, 2011). We follow the Cochrane Handbook recommendations for studies that do not correct for one of more of these type of errors, but a summary of how we will treat the results subject to various manifestations of unit of analysis errors is provided below.

- Cluster randomized trials:
  a. For cluster randomized trials that do not adjust for clustering in their results, given number of clusters, outcome data, and an estimate of ICC, we will adjust the analyses by dividing the reported sample size by the design effect,
1+(M−1)ICC,
  where *M* is the average cluster size and *ICC* is the intraclass correlation coefficient. If average cluster size and/or intraclass correlation is not provided in the paper, we will exclude the results of that paper (Rao & Scott, 1992).
- Cross‐over trials:
  a. While results from cross‐over trials that incorrectly specify the unit of analysis are problematic, we will include these results in analyses without adjustment because results suffering from such errors tend to be more conservative than the correct analyses.
- Repeated observations on participants:
  a. If data from multiple follow‐up rounds are reported, we will choose the follow‐up results that are furthest in time from the time of implementation.
- Events that may re‐occur:
  a. Study results that may be subject to counting events rather than participants, when patients should be the unit of analysis, and do not report on data of total number of patients which have experienced an event, will be excluded.
- Multiple treatment attempts:
  a. Studies for which the number of attempts at an intervention rather than the unit of randomization is used to produce results will use the same method as cluster randomized trials to correct for potential error.
- Multiple intervention groups:
  a. Studies with multiple intervention groups that are comparable, will report the results of one arm if it is indicated by study authors that this is the primary arm of interest for their analysis. Otherwise, we will combine the results of both arms into a single pair‐wise comparison.

#### 3.4.2 Criteria for determination of independent findings

In cases where a singular study provides results on more than one of our outcomes of interest, we will present each result separately. Similarly, if a singular study presents several measures for the same outcome, all results will be presented for completeness, but will be grouped by comparability of the effect size to other effect sizes, as categorized above. In addition, where multiple results for the same outcome of interest measured in the same way are presented within the same paper but are the result of different models/subgroup analyses, one effect size will be reported based on (a) the authors’ indication that the effect is one of the primary results of the study, or (b) the comparability of the effect size to other studies in the same group, as categorized using the characteristics listed above. If multiple articles are identified that report on the results from the same intervention, draw from the same study population and report on the same or very similar outcomes, we will report the effects from the earliest published article (Wilson, 2018). In each case, the authors will only report results that include our intervention/exposure and outcomes listed above.

#### 3.4.3 Meta‐analysis of primary outcomes

Primary analyses will focus on the primary outcomes listed above to analyse the effects of a given gender barrier‐targeted intervention/exposure on a given education outcome. For each study, we will assess whether any effect sizes are independent, dependent, or are the result of clustered analyses. If there are more than two studies grouped by the criterion above and all effect sizes within each group are assessed to be independent (e.g., not drawn from the same sample, not comparing multiple treatments with a common control, etc.), we will conduct a bivariate meta‐analysis using the metareg command in Stata 15 (Valentine, Pigott, & Rothstein, 2010); Harbord & Higgins, 2008). A method‐of‐moments random effects meta‐analysis will be used for these results, which is a generalization of the DerSimonian and Laird method (DerSimonian & Laird, 1986). If there are at least 10 studies in a given group and there exist effect sizes that are dependent (e.g., drawn from the same sample), we will run a bivariate random effects meta‐analysis with a robust variance estimator (Tanner‐Smith, & Tipton, 2014). Further, if there are at least 10 studies in a given group and there is clustering present in any given study's analysis, we will run a bivariate hierarchical meta‐analysis with a robust variance estimator (Tanner‐Smith, & Tipton, 2014). Meta‐analyses with dependent and clustered effect sizes will be run using the robumeta command in Stata 15 (Hedberg, 2011). Measures of between‐study variability will be reported in the form of the *Q*‐statistic, *I* ^2^ and *τ* ^2^.^9^ We will use forest plots to display study‐level and overall effect sizes.

#### 3.4.4 Meta‐analysis of secondary outcomes

We will also conduct a meta‐analysis of the results of secondary outcomes, following the same methodological decisions as to the type of model to run, which statistical package to use, and so forth, as primary outcomes, detailed above. This part of the quantitative synthesis will focus on how each intervention/exposure affects which hypothesized pathways operate in effective interventions to eliminate, or mitigate the effects of, gender‐related barriers to schooling, as stated above. The results of any meta‐analyses of secondary outcomes will be presented after those for primary outcomes. We will use forest plots to display study‐level and overall effect sizes.

#### 3.4.5 Causal mechanisms/pathways

In addition, hypothesized mechanisms/pathways linking how an intervention affects a given education outcome may be described in some detail in the background, results and/or discussion sections of a study, but researchers may not perform more formal testing of whether an intervention had an effect on a given mechanism. Recognizing that these are a part of author‐hypothesized explanations of results, we will collate and present these hypothesized mechanisms both as a part of a narrative description of results, as well as in table form alongside quantitative results of primary outcomes. These author‐hypothesized explanations of results will not be quantitatively analysed by our study team.

#### 3.4.6 Moderator analyses

An explanation of results of any meta‐analyses will attempt account for the differences between each study, including the frequency and duration of exposure and the context of the interventions, as the effectiveness of interventions to remove gender barriers may depend on context. Thus, provided there are more than two effect sizes and there is a sufficient amount of heterogeneity for a given moderator, we will attempt to conduct moderator analyses that will include the following variables in metaregressions.

##### 3.4.6.1 Aggregate context variables

- Geographic region: For example, sub‐Saharan Africa/South Asia/East Asia/Middle East and North Africa/Latin America, Urban/Rural, and Low Income/Middle Income countries, as defined by the World Bank.
- Gender Parity Index during the year of the intervention (or nearest available year), as defined by the UNESCO Institute for Statistics (http://data.uis.unesco.org/).
- Primary completion rate of country during the year of the intervention (or nearest available year) as defined by the UNESCO Institute for Statistics.

##### 3.4.6.2 Study‐level variables

- Journal articles versus non‐journal articles.
- Time of exposure, for example, before/after 2000.

##### 3.4.6.3 Intervention components

- Single versus multicomponent interventions.
- Binary variables for types of components included.

Given that we expect a large number of multicomponent studies among our pool of included studies, we are especially interested in the effect of different components on variability. Moderator analyses will also utilize method‐of‐moments random effects regressions using the metareg command in Stata 15 (Harbord & Higgins, 2008). Study data used in moderator analyses will be analysed using the intervention/exposure and outcome grouping framework stated above.

#### 3.4.7 Sensitivity analyses

As a part of sensitivity analyses, REML random effects model results of all applicable meta‐analyses and metaregressions will also be presented to assess whether results hold under a different calculation of the between‐study estimation component of *τ* ^2^ (Veroniki et al., 2016).

#### 3.4.8 Assessment of publication and selection bias

In order to assess the extent to which there may be publication and selection bias present, given a sufficient number of studies per intervention/exposure and outcome, we will include:

1. A funnel plot, noting possible reasons for asymmetry if it is present.
2. The trim‐and‐fill procedure to adjust the mean effect size.

### 3.5 Treatment of qualitative research

We do not plan to include purely qualitative research.

## ROLES AND RESPONSIBILITIES

● Content: Barbara Mensch, Stephanie Psaki
● Systematic review methods: Barbara Mensch, Stephanie Psaki, Erica Chuang
● Statistical analysis: Erica Chuang
● Information retrieval: Erica Chuang, Meredith Kozak and/or TBD project staff
● Advisory group members: David Evans (World Bank), Matthew Jukes (RTI International), Cynthia Lloyd (Independent Consultant), Patrick McEwan (Wellesley College), Birte Snilstveit (3ie)
● Other independent consultants and advisors: Katherine Willson (Independent Consultant), David Wilson (George Mason University)

## SOURCES OF SUPPORT

This project is funded by Echidna Giving.

## DECLARATIONS OF INTEREST

The review team has no known conflicts of interest.

## PRELIMINARY TIMEFRAME

● Literature search: October–November 2018
● Selection of studies, risk of bias assessment, and data extraction: November 2018‐June 2019
● Analysis: April–August 2019
● Submission: August–September 2019

## PLANS FOR UPDATING THE REVIEW

The authors plan to update the review within five years from the date of publication contingent on available funding.

## Footnotes

1 Note that, although a common intervention approach, poverty reduction interventions targeting girls will not be included in this review because there have been several recent systematic reviews that have analysed these programs (see, e.g., Baird, Ferreira, Özler, & Woolcock, 2014; Snilstveit et al., 2015).

2 Endogeneity arises when an explanatory variable is correlated with the error term either because it is unobserved/unmeasured, or because there is measurement error, or because there is simultaneity, that is, it is jointly determined with the dependent variable (Woolridge, 2018).

3 The list is drawn from Shadish, Cook, and Campbell (2002).

4 Studies that analyse the effects of a RCT but only report data collected after the intervention was implemented are eligible only if satisfy one of the following criteria: (a) studies do not suffer from issues of statistical power as given by sample size calculations reported in the study, or (b) they report a total sample size of over 10,000, or have more than 1,000 observations per cluster in each arm on average. These criteria are roughly drawn from Muralidharan (2017).

5 Note that, although a common intervention approach, poverty reduction interventions targeting girls will not be included in this review because there have been several recent systematic reviews that have analysed these programs (see, e.g., Baird et al. 2014; Snilstveit et al., 2015).

6 It is possible that there are effect sizes for which the equations provided may not be sufficient for conversion. In such cases, we will seek the guidance of a meta‐analysis methods expert to assist us in identifying the appropriate conversion equation(s).

7 For OLS and 2SLS models with continuous dependent variables, we strongly prefer using Equations 1.1–1.3, as we recognize that these are more direct estimates of partial correlations. However, in the absence of information from either the articles or from study author(s), we will use Equation 1.4).

8 For example, we will not use the same conversion equation for the results of an OLS models with a continuous independent variable and a continuous dependent variable and those from a logit or probit model.

9 We note that there are limitations to these measures when there are a small number of studies (Lipsey & Wilson, 2001).

## APPENDIX A

***Section A. Search Strategy and Search Terms***

1. Search Strategy

We will use the following search strategy to search ERIC and adapt it for the databases listed above. Searches will be limited to 2000‐2019 publication dates. The search terms below are grouped by:

- Geographic (regions and countries) Set (S4)
- Year Published Set (S5)
- Gender Set (S7)
- Education Set (S9)
- Study Set (S13)
- Intervention Set (S19)

2. Search Terms

**Table:**

S#	SET NAME	QUERY: BOOLEAN/PHRASE IN ERIC ON EBSCOHOST MONDAY FEBRUARY 11, 2019
S1	REGION	Africa OR Asia OR Caribbean OR ‘Central America’ OR ‘Latin America’ OR ‘Middle East’ OR ‘South America’ OR ‘West Indies’
S2	COUNTRIES	Afghanistan OR Albania OR Algeria OR Angola OR Argentina OR Armenia OR Azerbaijan OR Bangladesh OR Benin OR Belarus OR Belize OR Bhutan OR Bolivia OR Bosnia OR Herzegovina OR Botswana OR Brasil OR Brazil OR ‘British Virgin Islands’ OR Bulgaria OR ‘Burkina Faso’ OR Burundi OR ‘Cabo Verde’ OR ‘Cape Verde’ OR Cambodia OR Cameroon OR ‘Central African Republic’ OR Chad OR Chile OR China OR Colombia OR Comoros OR Congo OR ‘Costa Rica’ OR ‘Cote d’Ivoire’ OR ‘Ivory Coast’ OR Croatia OR Cuba OR ‘Czech Republic’ OR ‘Slovakia’ OR Djibouti OR Dominica OR ‘Dominican Republic’ OR ‘DPR Korea’ OR ‘East Timor’ OR ‘Timor Leste’ OR Ecuador OR Egypt OR ‘El Salvador’ OR Eritrea OR Estonia OR Ethiopia OR Fiji OR Gabon OR Gambia OR Gaza OR (Georgia W5 republic) OR Ghana OR Grenada OR Guatemala OR Guinea OR ‘Guinea Bissau’ OR Guyana OR Haiti OR Honduras OR Hungary OR India OR Indonesia OR Iran OR Iraq OR Jamaica OR Jordan OR Kazakhstan OR Kenya OR Kiribati OR Kosovo OR ‘Kyrgyz Republic’ OR Kyrgyzstan OR ‘Lao PDR’ OR Laos OR Latvia OR Lebanon OR Lesotho OR Liberia OR Libya OR Lithuania OR Macedonia OR Madagascar OR Malaysia OR Malawi OR Maldives OR Mali OR ‘Marshall Islands’ OR Mauritania OR Mauritius OR Mexico OR Micronesia OR Moldova OR Mongolia OR Montenegro OR Morocco OR Mozambique OR Myanmar OR Burma OR Namibia OR Nauru OR Nepal OR Nicaragua OR Niger OR Nigeria OR ‘Northern Mariana Islands’ OR Oman OR Pakistan OR Palau OR Panama OR Paraguay OR Peru OR Philippines OR Poland OR Romania OR ‘Russian Federation’ OR Russia OR Rwanda OR ‘Saint Kitts’ OR ‘St Kitts’ OR Nevis OR ‘St Lucia’ OR ‘Saint Lucia’ OR ‘Saint Martin’ OR ‘St Martin’ OR ‘St Vincent’ OR ‘Saint Vincent’ OR Grenadines OR Samoa OR ‘Sao Tome’ OR Principe OR Senegal OR Serbia OR Seychelles OR ‘Sierra Leone’ OR ‘Soloman Islands’ OR Somalia OR ‘South Africa’ OR ‘Sri Lanka’ OR Sudan OR Suriname OR Swaziland OR ‘Syrian Arab Republic’ OR Syria OR Tajikistan OR Tanzania OR Thailand OR Togo OR Tonga OR Trinidad OR Tobago OR Tunisia OR Turkey OR Turkmenistan OR (Turks W1 Caicos) OR Tuvalu OR Uganda OR Ukraine OR Uruguay OR Uzbekistan OR Vanuatu OR Venezuela OR Vietnam OR ‘Viet Nam’ OR ‘West Bank’ OR Yemen OR Zambia OR Zimbabwe
S3	LMIC TEXTWORDS	(developing W1 nation) OR (developing W1 nations) OR (developing W1 countr*) OR (developing W1 econom*) OR (developing W1 world) OR (less* W1 developed W1 nation) OR (less* W1 developed W1 nations) OR (less* W1 developed W1 countr*) OR (less* W1 developed W1 econom*) OR (less* W1 developed W1 world) OR (under* W1 developed W1 nation) OR (under* W1 developed W1 nations) OR (under* W1 developed W1 countr*) OR (under* W1 developed W1 econom*) OR (under* W1 developed W1 world) OR (underdeveloped W1 nation) OR (underdeveloped W1 nations) OR (underdeveloped W1 countr*) OR (underdeveloped W1 econom*) OR (underdeveloped W1 world) OR (low* W2 income W1 nation) OR (low* W2 income W1 nations) OR (low* W2 income W1 countr*) OR (low* W2 income W1 econom*) OR (middle W1 income W1 nation) OR (middle W1 income W1 nations) OR (middle W1 income W1 countr*) OR (middle W1 income W1 econom*) OR (underserved W1 nation) OR (underserved W1 nations) OR (underserved W1 countr*) OR (deprived W1 nation) OR (deprived W1 nations) OR (deprived W1 countr*) OR (poor W1 nation) OR (poor W1 nations) OR (poor W1 countr*) OR (third W1 world) OR (transitional W1 nation) OR (transitional W1 nations) OR (transitional W1 countr*) OR (transitional W1 econom*)
S4	GEOGRAPHIC SET	S1 or S2 OR S3
S5	YEAR PUBLISHED	YR 2000‐2019
S6	GEO/YR SET	S4 AND S5
S7	GENDER SET	gender OR woman OR women OR female OR females OR girl* OR (sex W1 role*) OR (sex W1 difference*) OR DE sex differences OR DE sex role
S8	GEN/GEO/YR SET	S6 AND S7
S9	EDUCATION SET	SU education* OR school* OR learn*
S10	ED/GEN/GEO/YR SET	S8 AND S9
S11	STUDY DESIGN	(‘random* control* trial*’) OR (control W1 endogeneity) OR (regression W1 discontin*) OR (regression W1 model*) OR (‘instrumental variable* analys*’) OR (‘interrupted time series’) OR (pre W1 test*) OR (post W1 test*) OR (pretest*) OR (posttest*) OR (‘match* comparison group*’) OR (matching N2 procedure*) OR (comparison W1 group*) OR (control W1 group*) OR (quasi W1 experiment*) OR (quasiexperiment*) OR (mixed W1 method*) OR (cross W1 random*) OR (least W1 square* W1 model*) OR (experiment*) OR SU regression OR DE randomized controlled trials OR DE pretests posttests OR DE quasiexperimental design OR DE educational experiments OR DE educational research OR DE mixed methods research OR DE program effectiveness OR DE program evaluation
S12	STUDY RESULT	(evidence N4 intervention*) OR (evaluat* N4 intervention*) OR (impact* N4 intervention*) OR (effect* N4 intervention*) OR (result* N4 intervention*) OR (outcome* W1 measure*) OR (causal) OR (case W1 stud*) OR (best W1 practice*) OR (what W1 workS) OR (program W2 result*) OR (program W1 evaluation*) OR (program W1 effect*)
S13	STUDY SET	S11 OR S12
S14	STUDY/ED/GEN/GEO/YR SET	S10 AND S13
S15	INTERVENTION DESCRIPTORS	DE academic achievement OR DE access to education OR DE after school programs OR DE critical thinking OR DE curriculum OR DE daily living skills OR DE educational attainment OR DE empowerment OR DE emotional development OR DE fees OR DE HIV OR DE AIDS OR DE marriage OR DE married students OR DE pregnancy OR DE pregnant students OR DE sanitary facilities OR DE sanitation OR DE school safety OR DE school security OR DE school uniforms OR DE sexually transmitted infections OR DE student costs OR DE teacher education OR DE textbooks OR DE violence OR DE women administrators OR DE women faculty
S16	INTERVENTIONS TEXTWORDS	(academic W1 achieve*) OR (academic W1 engagement) OR (grade W1 attain*) OR (grade* N2 repetition) OR (grade* N2 progress*) OR (education* W1 attain*) OR (enroll* N2 status) OR (school* N2 access) OR (school * W1 participation) OR (school* W1 supplies) OR (school* N3 fee*) OR (school* N3 dropout*) OR (school* N2 facilit*) OR (school* N2 infrastructur*) OR (school* N4 sanita*) OR (school* N2 quality) OR (school* N2 complet*) OR (school* N2 enroll*) OR (school* N2 continu*) OR (school* N2 attend*) OR (school* N2 safety) OR (school* W1 security) OR (school* N4 uniform*) OR (school* N4 (toilet* OR latrine*)) OR (village W2 school*) OR (distance W2 school*) OR (barrier* N3 retention) OR (gender n4 violence) OR (gender wN2 sensitiv*) OR (gender W1 norm*) OR (gender N2 discriminat*) OR (gender N3 attitude*) OR (early W1 childbearing) OR (early W1 marr*) OR (child W1 marr*) OR (adolescen* N2 pregnan*) OR (adolescen* N2 childbearing) OR (adolescen* W1 mother*) OR (parent* N3 attitude*) OR (opportunity W1 cost*) OR (direct W1 cost*) OR (legal W1 framework*) OR (political N2 support) OR (health W2 issue*) OR (HIV N3 prevent*) OR (STI OR STD) OR (cash W1 transfer*) OR (access W2 service*) OR (transition N2 work*) OR (teacher N3 bias*) OR (teacher N3 attitude*) OR (teacher N3 train*) OR (teacher W1 education) OR (female* N3 administrator*) OR (female* N3 teach*) OR (years W2 school*) OR (gender N2 equality) OR (earning N2 capacity) OR (student* N4 pregnan*) OR (curricul*) OR (women W1 faculty) OR (women W1 administrat*) OR (student W1 cost*) OR (after W1 school W1 program*) OR (married W1 student*) OR (textbook*) OR (menstrual) OR (student* N3 re‐enrollment) OR (test score*) OR (exam taking) OR (sexual W1 reproductive W1 health)
S17	SKILL INTERVENTION TERMS	(critical W1 thinking) OR (emotion al W1 learning) OR (daily W1 living) OR (emotional W1 development) OR (self W1 efficacy) OR (mental W1 health) OR (knowledge W1 rights) OR (civic W1 engagement) OR (financial W1 literacy) OR (self W1 aware*) OR (skill* N2 acqui*) OR (cognitive W1 ability)
S18	SKILLS INTERVENTION GROUPED	(life OR negotiat* OR relationship* OR decisionmaking OR agency OR empower* OR abuse OR ambition OR coercion OR communicat* OR autonomy OR literacy OR numeracy OR advocacy OR resilience OR harassment OR aspiration OR sexual*) N5 skill*
S19	INTERVENTION SET	S15 OR S16 OR S17 OR S18
S20	INTERV/STUDY/ED/GEN/GEO/YR SET	S14 AND S19

***Section B. Assessment of Risk of Bias***

The following risk of bias tools were adapted from RoB 2 (Higgins et al., 2016) for randomized studies, and ROBINS‐I (Sterne et al., 2016) and Psaki et al. (2019) for nonrandomized studies. The following are the values for response options:

- Y = “Yes”
- PY = “Possibly Yes”
- PN = “Possibly No”
- N = “No”
- NI = “No Information”
- NA = “Not Applicable”

Values in green indicate response options for which there may be low risk of bias. Values in red indicate response options for which there may be high risk of bias. We follow the guidelines for RoB 2, ROBINS‐I and Psaki et al. (2019) as closely as possible to judge for risk of bias.

1) Randomized Studies

**Table:**

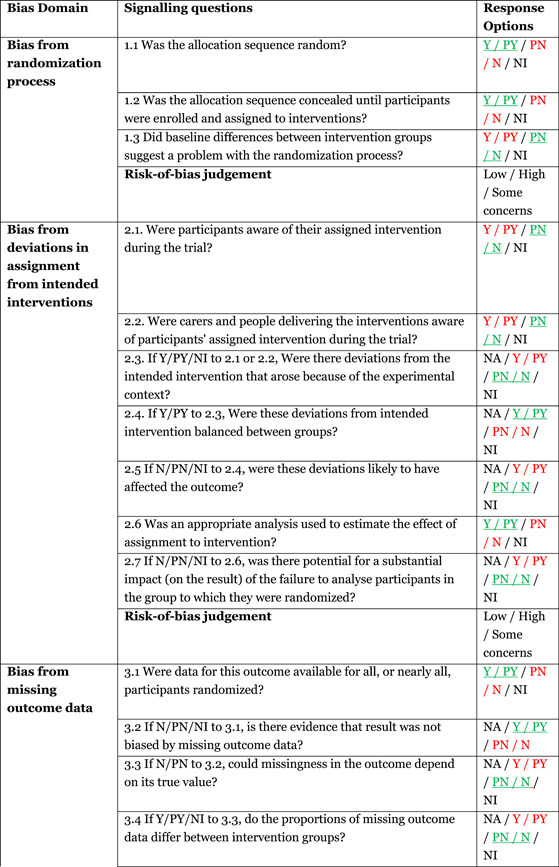

**Table:**

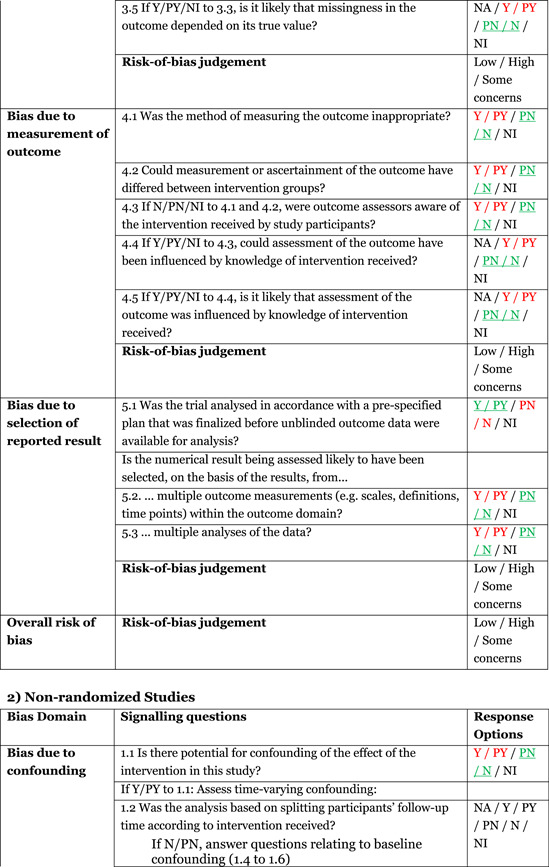

**Table:**

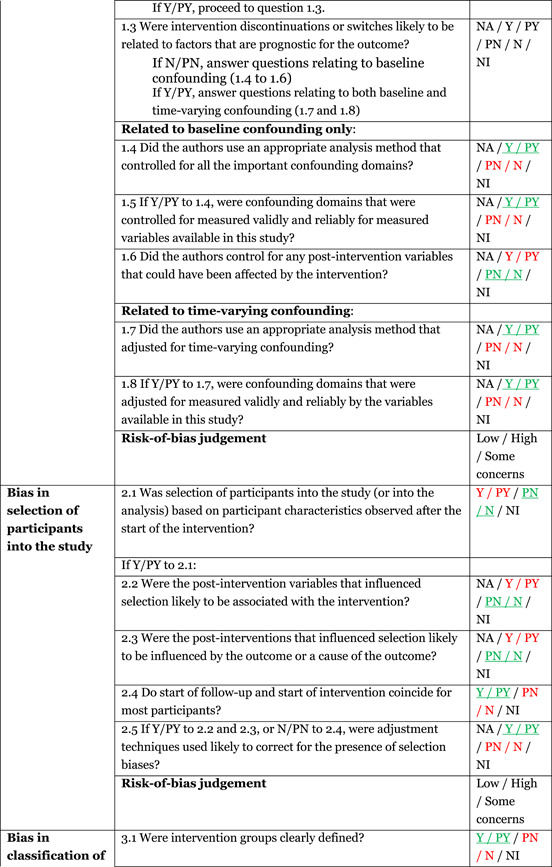

**Table:**

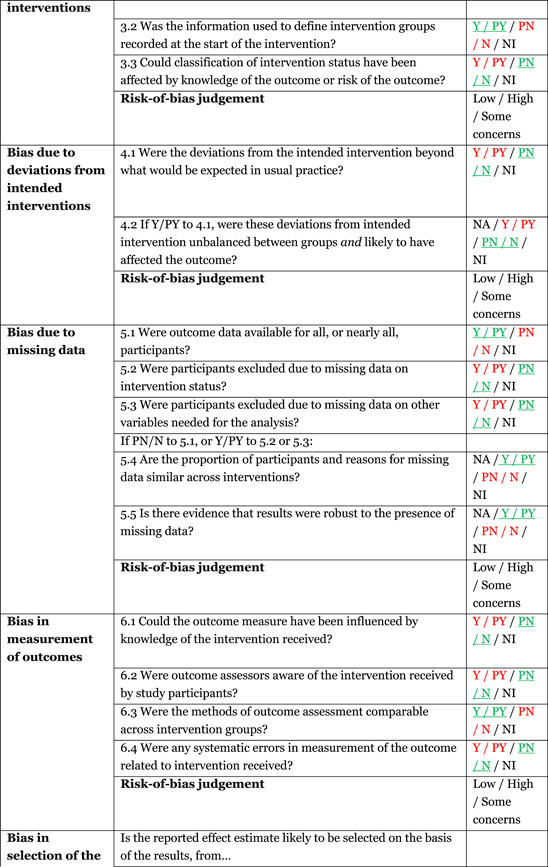

**Table:**

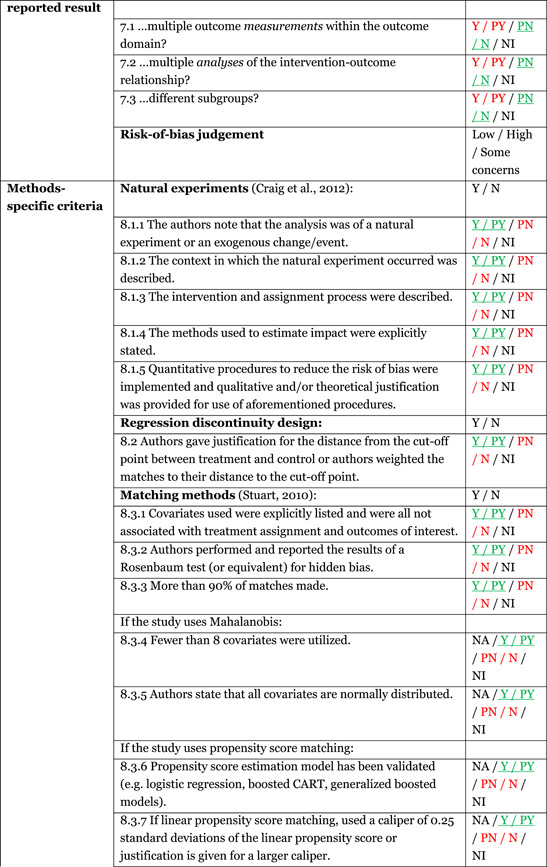

**Table:**

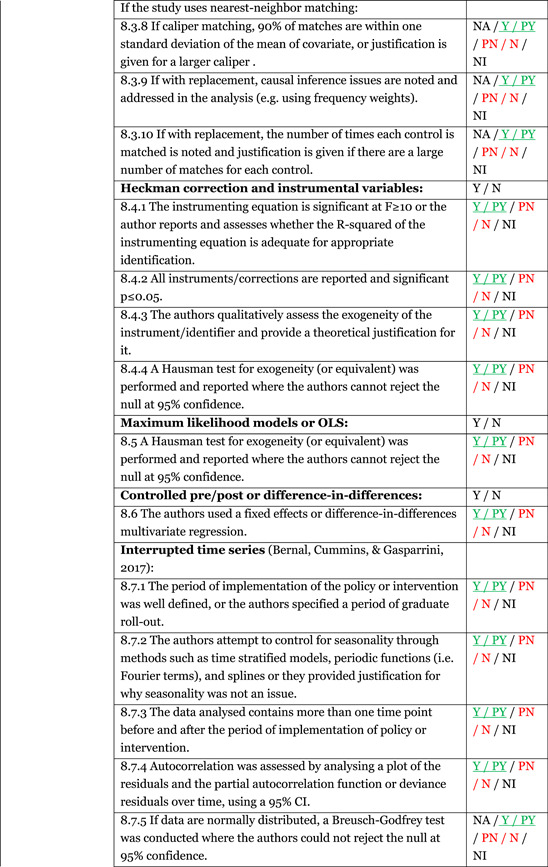

**Table:**

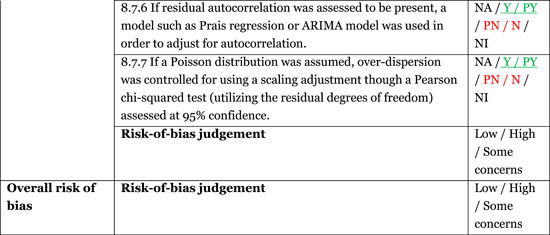

***Section C. Conversion Equations***

Note: IV here refers to independent variable, rather than instrumental variable. DV refers to the dependent variable. For any two‐stage regressions, the second stage regressor is referenced as the IV. r represents the partial correlation.

1. **Continuous Dependent Variables:**

- **OLS or 2SLS Models with Either Continuous or Dichotomous IVs**
  ○ Equation 1.1:
    ▪ $r=\frac{{\mathrm{SD}}_{x}B}{{\mathrm{SD}}_{y}}$
    ▪ Data needed:
      ● Unstandardized Regression Coefficient (B)
      ● Standard deviation of DV ${(\mathrm{SD}}_{y})$
      ● Standard deviation of IV ${(\mathrm{SD}}_{x})$
  ○ Equation 1.2, if only ${\mathrm{SD}}_{y}$ is provided:
    ▪ $d=\frac{B}{{\mathrm{SD}}_{y}}$, where d refers to Cohen's *d*
    ▪ $r=\frac{d}{\sqrt{4+d^{2}}}$
    ▪ Data needed:
      - Unstandardized Regression Coefficient (B)
      - Standard deviation of DV ${(\mathrm{SD}}_{y})$
  ○ Equation 1.3, if the SD of either x or y are reported separately by treatment and control groups:
    ▪ $\mathrm{SD}=\sqrt{\frac{{\mathrm{SD}}_{t}^{2}\left(n_{t}-1\right)+{\mathrm{SD}}_{c}^{2}\left(n_{c}-1\right)+\left(\frac{{\overset{®}{x}}_{t}-{\overset{®}{x}}_{c}}{2}\right)\;(n_{t}+n_{c})}{n_{t}+n_{c}-1}}$
    ▪ $r=\frac{{\mathrm{SD}}_{x}B}{{\mathrm{SD}}_{y}}$
    ▪ Data needed:
      ● Unstandardized Regression Coefficient (B)
      ● Standard deviation of either DV or IV for treatment group ${(\mathrm{SD}}_{t})$
      ● Standard deviation of either DV or IV for control group ${(\mathrm{SD}}_{c})$
      ● Mean of treatment group ${(\overset{®}{x}}_{t})$
      ● Mean of control group ${(\overset{®}{x}}_{c})$
      ● Treatment group sample size ($n_{t}$)
      ● Control group sample size ($n_{c}$)
  ○ Equation 1.4, if only *B* and ${\mathrm{se}}_{B}$ are provided:
    ▪ Equations:
      1. $t=\frac{B}{{\mathrm{se}}_{B}}$, where t refers to the t‐statistic
      2. $r=\frac{t}{\sqrt{t^{2}+\mathrm{df}}}$
    ▪ Data needed:
      ● T‐statistic (t) or Unstandardized Regression Coefficient and Standard Error ($B,{\mathrm{se}}_{B}$)
      ● Residual Degrees of Freedom (sample size minus the number of predictors) (df)

2. **Dichotomous Dependent Variables**

- **Logit Models**
  ○ Equation 2.1: Logit models with dichotomous IV and dichotomous DV
    ▪ Equations:
      1. B=log⁡(OR)
      2. $d=B(\frac{\sqrt{3}}{\pi })$
      3. $r=\frac{d}{\sqrt{4+d^{2}}}$
    ▪ Data needed:
      ● Unstandardized Regression Coefficient or Odds Ratio (B or OR)
- **OLS or 2SLS Models with Dichotomous Independent Variables**
  ○ Equation 2.2.1: OLS or 2SLS models with dichotomous IV and dichotomous DV (if control group success proportion is presented)
    ▪ Equations:
      1. $a=n_{t}(p_{c}+B)$
      2. $b=n_{t}(1-(p_{c}+B))$
      3. $c=n_{c}⁎p_{c}$
      4. $d=n_{c}(1-p_{c})$
      5. $r=\frac{\left(\mathrm{ad}\right)-(\mathrm{bc})}{\sqrt{\left(a+b)(c+d)(a+c)(b+d\right)}}$
    ▪ Data needed:
      ● Unstandardized Regression Coefficient (B)
      ● Treatment group sample size ($n_{t}$)
      ● Control group sample size ($n_{c}$)
      ● Control group success proportion (i.e. mean) of DV ($p_{c}$)
  ○ Equation 2.2.2: OLS or 2SLS with dichotomous IV and dichotomous DV (if only overall success proportion is presented)
    ▪ Equations:
      1. $a=n_{t}(p+.5B)$
      2. $b=n_{t}(1-(p+.5B))$
      3. $c=n_{c}(p-.5B)$
      4. $d=n_{c}(1-(p-.5B))$
      5. $r=\frac{\left(\mathrm{ad}\right)-(\mathrm{bc})}{\sqrt{\left(a+b)(c+d)(a+c)(b+d\right)}}$
    ▪ Data needed:
      ● Unstandardized Regression Coefficient (B)
      ● Treatment group sample size ($n_{t}$)
      ● Control group sample size ($n_{c}$)
      ● Overall success proportion (i.e. mean) of DV (p)

3. **Standard Errors and Confidence Intervals**

- **Standard Errors**
  ○ If only the standard error of the coefficient is available:
    ▪ Equation 3.1:
      ● ${\mathrm{se}}_{r}=\frac{r⁎{\mathrm{se}}_{B}}{B}$, where ${\mathrm{se}}_{r}$ refers to the standard error of the Partial correlation
    ▪ Data needed
      ● Unstandardized Regression Coefficient (B)
      ● Standard Error of the Unstandardized Regression Coefficient (${\mathrm{se}}_{B}$)
  ○ If only the 95% confidence intervals for the coefficient are available:
    ▪ Equation 3.2:
      1. ${\mathrm{se}}_{B}=\frac{{\mathrm{CI}}_{\mathrm{upper}}-{\mathrm{CI}}_{\mathrm{lower}}}{1.96}$, where ${\mathrm{se}}_{B}$ refers to the standard error of the unstandardized regression coefficient
      2. ${\mathrm{se}}_{r}=\frac{r⁎{\mathrm{se}}_{B}}{B}$, where ${\mathrm{se}}_{r}$ refers to the standard error of the Partial correlation
    ▪ Data needed
      ● Unstandardized Regression Coefficient (B)
    ▪ Confidence intervals of the Unstandardized Regression Coefficient (${\mathrm{CI}}_{\mathrm{upper}}$, ${\mathrm{CI}}_{\mathrm{lower}}$)
- **Confidence Intervals**
  ○ The equation below can apply to either regression coefficients as well as partial correlations:
    ▪ Equation 3.3
      1. $\mathrm{CI}=B\pm {\mathrm{se}}_{B}∙1.96$
